# Person-centred care for people with tuberculosis-associated comorbidities: a multi-country qualitative study

**DOI:** 10.1136/bmjopen-2025-106529

**Published:** 2025-11-28

**Authors:** Stephanie Law, Annabel Baddeley, Anna Carlqvist, Farai Mavhunga, Kerri Viney, Amrita Daftary

**Affiliations:** 1 Dahdaleh Institute for Global Health Research, York University, Toronto, Ontario, Canada; 2 McGill International TB Centre, Research Institute of the McGill University Health Centre, Montreal, Ontario, Canada; 3 Department for HIV, Tuberculosis, Hepatitis and Sexually Transmitted Infections, World Health Organization, Geneva, Switzerland; 4 World Health Organization, Geneva, Switzerland; 5 Centre for the AIDS Programme of Research in South Africa, University of KwaZulu-Natal, Durban, South Africa

**Keywords:** Tuberculosis, Patient-Centered Care, Organisation of health services, Person-Centered Care, Multimorbidity, QUALITATIVE RESEARCH

## Abstract

**Abstract:**

**Introduction:**

To contribute to the development of a people-centred global framework for collaborative action on tuberculosis (TB) and comorbidities, a rapid qualitative study on the perspectives of people with lived experience of TB and its associated comorbidities was undertaken.

**Methods:**

From August to October 2021, TB survivors from high-burden countries, who encountered at least one comorbidity during TB treatment, were interviewed to explore their healthcare experiences and priorities. Thematic analysis drew on a healthcare acceptability model.

**Results:**

Participants (n=24, 13 women) were treated for drug-susceptible (n=13) or drug-resistant (n=11) TB between 2015 and 2021. They faced diverse comorbidities (mental health and substance use disorders, diabetes, Hepatitis C, lupus and HIV); half of whom reported more than one comorbidity, and all faced socioeconomic hardships. TB diagnosis and treatment exacerbated participants’ comorbidities and, in the absence of integrated support, precipitated mental health challenges. Four healthcare priorities for addressing TB-associated comorbidities were identified: (1) disclosure and early identification of comorbidities, (2) timely and affordable access to care for comorbidities, (3) tailored counselling and peer support and (4) coordinated and consolidated care for TB and comorbidities.

**Conclusion:**

The syndemic manifestation of comorbidities in people affected by TB calls for a people-centred approach to care that facilitates building of trust with multiple care providers, timely linkages to non-TB programmes, access to integrated diagnosis and treatment, allaying intersecting stigmas and self-shame, and care coordination approaches that correspond to people’s needs and preferences. These healthcare priorities were included in the WHO’s *Framework for collaborative action on TB and comorbidities*.

**STRENGTHS AND LIMITATIONS OF THIS STUDY**

- Our multi-country purposive sampling approach enabled the identification of priorities among people affected by tuberculosis (TB) that are relevant across all WHO regions, all genders and diverse comorbidities.
- Our small study sample (n=24) allowed initial exploration of convergent and divergent perspectives across WHO regions, but additional research is required to understand differences related to gender and health system contexts.
- Although our referral-based snowball sampling approach through community-based NGOs and research partners might over-represent individuals connected to specific TB service and survivor networks, it allowed us to effectively reach marginalised populations with stigmatising conditions like substance use and mental health issues by leveraging established trust relationships.

## Introduction

 Tuberculosis (TB) rarely occurs alone. Globally, up to two thirds of people with TB experience one or more chronic comorbidities.^1^ The most common comorbidities are depression, HIV and diabetes mellitus.^2^ Comorbidities interact synergistically with TB, amplifying disease burdens and worsening TB treatment outcomes.^3^,^5^ Efforts to address comorbidities have largely centred on addressing HIV-associated TB, where global uptake of collaborative TB/HIV activities has improved TB outcomes among people living with HIV, and vice versa.^6 7^ However, the integration of diagnostic and treatment services for other common TB-associated comorbidities, especially non-communicable diseases (NCDs), is rarely considered.^8^ Even when people with TB are screened for other comorbidities, they are seldom followed up with systematic linkages to care.^8 9^

The diversity of clinical and social nuances associated with TB and these comorbidities requires a people-centred approach. Such an approach should respond to potentially conflicting treatment demands, enhance continuity of care, strengthen collaboration across health sectors and providers, and improve health equity. According to the WHO, “people-centred services provide holistic, individualised, empowering and respectful care, organised around the comprehensive needs of the person rather than around individual diseases.”^10^ The need for a people-centred approach to treating and managing TB-related comorbidities is highlighted in WHO’s End TB Strategy^11^ and in the Political Declaration of the United Nations High Level Meeting on the fight against tuberculosis.^12^

However, despite these global calls, there has been slow and limited uptake of policies and interventions to address TB and related comorbidities. Thus, WHO recently developed a *Framework for collaborative action on TB and comorbidities* (‘Framework’) in consultation with experts and key stakeholders.^10^ The *Framework* aims to provide evidence-informed guidance for implementing and expanding people-centred services for TB and related comorbidities, targeting healthcare workers, policy and decision makers, researchers and non-governmental and civil society organisations, around the world. At the time of its development in 2021–2022, there was a paucity of primary research examining the healthcare perspectives and preferences of people who experienced comorbidities other than HIV during their TB treatment. Thus, we conducted the following qualitative study with TB survivors from high TB burden countries to fill this knowledge gap and inform the development of the *Framework*.

## Methods

### Recruitment

To inform stakeholder consultation meetings for developing the *Framework*, the study timeframe was short (2 months) and hence a pragmatic qualitative approach was necessary. We reached out to contacts and collaborators at TB-focused local, regional and global organisations, including community and civil society organisations, covering all six WHO regions. We informed them of the study and asked them to share it with their networks and refer potential eligible participants to us. Interested individuals either gave the recruiting organisation permission to share their contact information with us or contacted us directly. Participant inclusion criteria were: residing in a country on the WHO global lists of high burden countries for drug-susceptible TB (DS-TB) or multidrug/rifampicin-resistant TB (/RR-TB)^13^; received treatment in that country for any type of TB in the preceding 5 years (ie, from January 2015 to August 2021); and experienced at least one comorbidity during TB treatment. We focused on this period to align with the release of the 2015 WHO End TB Strategy^11^ and capture a diversity of TB survivor experiences. Persons aged under 18 years or receiving TB treatment at the time of recruitment were excluded. All eligible individuals who were approached accepted to participate in the study.

To balance achieving saturation on key general themes relevant to people-centred care in TB-associated comorbidities and the short study timeframe, we set the target sample size to 24, informed by prior qualitative research decisions.^14^ Under a purposive sampling framework, including referral-based and snowball sampling, we aimed to recruit 4–6 people with a history of underreported NCDs (diabetes, mental health and substance use including use of drugs, alcohol and/or tobacco), 4–6 people overall from regions with highest burdens of TB (Southeast Asia, Eastern Mediterranean, Africa) and 50% women.

### Data collection

**Figure 1:**
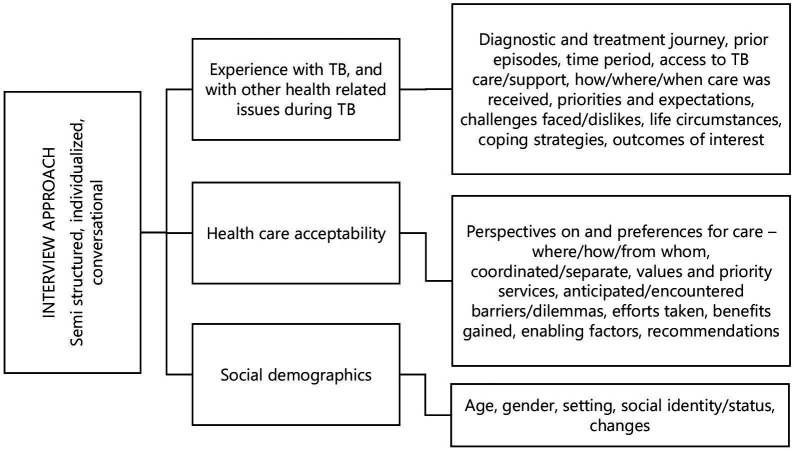
Summary of qualitative interview approach. TB, tuberculosis.

One-time interviews (45 to 90 min) with individuals who provided informed consent were conducted via phone, encrypted WhatsApp, Viber or Zoom, as per participants’ preferences, by an experienced postdoctoral qualitative researcher who identifies as an East Asian woman (SL). The interviewer had no prior interactions with participants and commenced each interview with a brief introduction of her role as a postdoctoral researcher and experience in conducting qualitative TB research. The in-depth, one-on-one interviews followed a semi-structured format^15^ to explore participant experiences with, and acceptability of care for, TB and comorbidities (figure 1). The interview guide (online supplemental material) drew from a healthcare acceptability framework developed by Sekhon *et al*,^16^ specifically inquiring on several dimensions of acceptability (affective attitude, burden, ethicality), and was informed by the study team’s robust experience in qualitative TB research (SL, AD) and policy development (AB, AC, FM, KV). During the interviews, the interviewer shaped open-ended questions relative to participants’ responses, aiming to understand their values and opinions, as well as underlying thought processes. The interviewer made field notes during and after each interview to facilitate reflexivity and iterative analysis. Interviews were conducted in English or, at participants’ request and with an interpreter’s assistance, Russian, Spanish or Urdu, and were audio-recorded and transcribed. In line with iterative qualitative practice, two researchers (SL, AD) met regularly to discuss field notes and preliminary reflections from interviews, identify perspectives that need to be further explored or were missing and determine additional purposive sampling needs, if necessary.^14 17^ We did not share interview transcripts with participants; however, two participants were contacted after their interviews to ascertain missing or unclear details related to their interviews (ie, timing of mental health problems and previous healthcare experiences).

### Analysis

We performed reflexive thematic analysis^18^ to inductively develop themes based on interview transcripts using Microsoft OneNote. Via multiple readings of the transcripts, one qualitative researcher (SL) generated initial codes to describe the data and created a coding tree (online supplemental material). Another researcher (AD) reviewed these initial codes and a selection of transcripts, suggested additional codes and discussed potentially divergent, initial interpretations of the data. The researcher (SL) then iteratively compared, collapsed and related the codes to develop themes—paying close attention to similarities and differences in experiences based on the participant’s sex, comorbidity and geopolitical context—and created a visual model to conceptualise the themes. In lieu of participant checking, the researchers (SL, AD) practised reflexivity throughout by interrogating and bracketing their own values, presumptions and perspectives.^19^ Subsequent discussions with the full study team enhanced interpretative confirmability and helped finetune the themes and visual model.^20 21^ Reporting of this study adheres to the Consolidated criteria for reporting qualitative research (COREQ) 32-item checklist.^19^

### Patient and public involvement

TB-focused local, regional and global organisations, including community/civil society organisations and patient advocacy organisations, aided in study recruitment by referring potential participants. Patients and the public were not involved in any other aspect of the study (ie, design, conduct other than recruitment, reporting or dissemination).

## Results

### Overview

Participants included 13 women and 11 men (n=24) (table 1) who started TB treatment between 2015 and 2021 in 11 countries (Georgia, India, Indonesia, Kazakhstan, Kenya, Moldova, Pakistan, Peru, the Philippines, South Africa and Ukraine). Comorbidities included: diabetes mellitus, hepatitis C (HCV), HIV, lupus, mental health conditions and tobacco/substance use; half (n=12) reported more than one comorbidity during TB treatment. To protect their anonymity, we report only their TB types and comorbidities alongside illustrative quotes.

**Table 1:** Study participants (n=24)

Characteristic	N (% or range)
Female	13 (54.1)
Median age in years	37 (21–60)
Type of comorbidity	
HIV	6 (25.0)
Diabetes	5 (20.8)
Mental health*	14 (58.3)
Tobacco/substance use†	8 (25.0)
Other‡	4 (29.2)
More than one comorbidity	12 (50.0)
Type of TB	
DS-TB	13 (54.2)
RR/MDR-TB	11 (45.8)
Start year of TB treatment	2015–2021
WHO region	
African	4 (16.7)
Americas	2 (7.7)
Eastern Mediterranean	6 (25.0)
Europe	6 (25.0)
South-East Asia	5 (20.8)
Western Pacific	1 (4.2)

* Reported as depression, anxiety, panic, hallucinations, suicidal thoughts and/or emotional or nervous breakdown.

† Reported substances included alcohol, cannabis, heroin and other unspecified drugs.

‡ Other comorbidities included HCV and lupus.

DS-TB, drug-susceptible TB; HCV, hepatitis C virus; RR/MDR-TB, rifampicin-resistant/multidrug-resistant TB; TB, tuberculosis.

Participants described many socioeconomic and biomedical challenges during TB treatment, including unmanaged symptoms and side effects, social isolation, stigma and financial hardship. These challenges, which presented as a common backdrop against which participants experienced TB and its treatment, were exacerbated by their comorbidities, and vice versa. For example, participants with diabetes struggled to maintain a low glycaemic diet while fending off the side effects of TB medication.

I knew my treatment [for diabetes], I knew the advice, but I didn’t have the means to do it. Maybe I could [be] disciplined on the things that I am eating, but on the other hand, I need to eat also so that I can consume the 17 [TB] medicines that I am taking in a day. It was ironic that I have to control my [blood] sugar so that I can be well, but then I have to eat something after taking my [TB] meds so that I will not vomit the medicine I am taking. –MDR/RR-TB, diabetes

Most reported comorbidities preceded TB diagnosis, except for mental health conditions, including suicidal ideation, which many participants attributed to TB and its associated burden. Among those with a history of substance use, unmanaged mental health conditions additionally heightened the risk of relapse.

There was even a time when I started [drinking] alcohol…I knew drinking wasn’t good when it comes to TB drugs, but I did it because of the stigma; why should I live in this world if my own family doesn’t want me? –DS-TB, mental health, substance use & HIV

**Figure 2:**
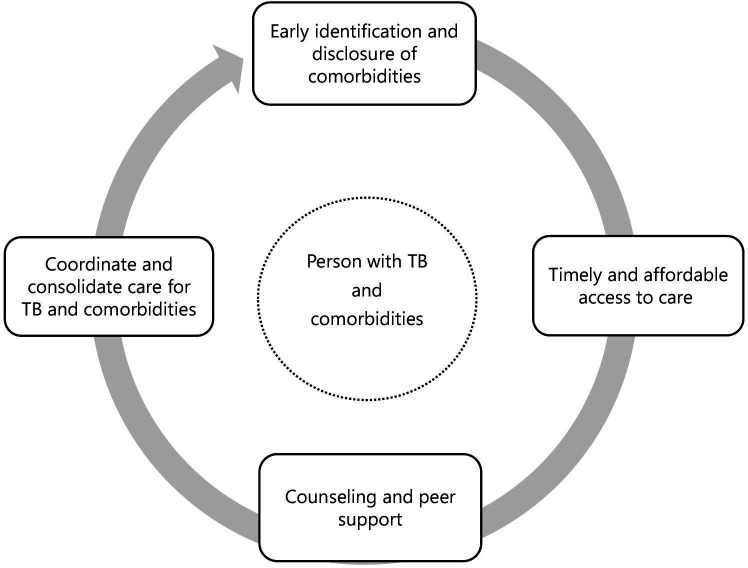
Priorities for a person-centred continuum of care for people with TB and comorbidities. TB, tuberculosis.

Participants’ reflections about their experiences, needs and values enabled the identification of four cross-cutting gaps that were relevant across diverse settings and comorbidities. These gaps populated priority themes for developing a person-centred continuum of care for people affected by TB and comorbidities (figure 2). We describe each theme, including illustrative quotes, in the following sections.

### Disclosure and early identification of comorbidities

Participants understood TB care providers should have a ‘full picture’ of their health, but their non-HIV comorbidities were seldom assessed. Instead, identification depended largely on proactive self-disclosure, which in turn depended on perceived value, stigma and risks of disclosure.

They would ask me these questions, ‘did you feel suicidal?’ I would say no…I would often lie. I wouldn’t let them know about the severity of what I was going through. I feared if I told them honestly, I would be put in a psychiatric ward or put on some [more] medications. – MDR/RR-TB, mental health

Thus, a universal request was for TB care providers to clearly explain the medical relevance of identifying comorbidities, avoid fear-mongering and stigmatising language and display empathy over judgement.

It really matters how the doctor asks the question, what words he would use, and how he would show his attitude. If the doctor asked directly, ‘are you a drug addict?’ Then I would say no, I’m not. If he would show no prejudice or blame, and that he needs this information in order to help better, then that would make a difference. – DS-TB, substance use, HCV & mental health

Participants who hid comorbidities and related problems from their TB care providers opened up only after repeat queries over time, during which a trusting relationship developed. One participant was afraid to share that he could no longer afford diabetes medication and was distressed over his inability to support his family.

At the beginning, I would not look [the psychologist] in the eyes, and I would try to make them believe everything was fine…I didn’t open up because I had a mentality that the psychologist study you, so I wasn’t willing to open up. But she dug, so I opened up and spoke about it a couple of times.—DS-TB, diabetes & mental health

On opening up, this participant started receiving the care needed for both his diabetes and psychological state. Several participants suggested home visits from discrete, sensitive providers and meetings with peer counsellors could help uncover issues that are stigmatising or embarrassing to discuss at TB facilities such as substance use and depressive feelings.

### Timely and affordable access to care for comorbidities

Participants faced diverse access barriers to comorbidity care, ranging from a lack of prioritisation—by both themselves and their TB care providers—to prohibitive medication costs. As with disclosure, some participants were not forthcoming with access problems to their care providers, which led to further delays and potentially negative consequences.

If [the providers] would have asked me, ‘how are you?’, of course I would have told them this is my experience, that sometimes I cannot afford this and that…or maybe they would know that [I am] not controlling [my blood] sugar…but no, nobody is asking, so I didn’t bother to talk to them. –MDR/RR-TB, diabetes

For participants with mental health conditions or a known history of substance use, access might be guarded or even refused by dismissive providers.

I felt really depressed…but the family doctor was very reluctant to prescribe any medicine because [the city has] high drug use, so [doctors] are suspicious of people who are asking for medicines for depression because they really want to buy some drugs. So, they’re reluctant, they don’t take it seriously, they say, ‘it doesn’t matter if you’re depressed, you can grow calm.’ That’s all, they don’t care. – MDR/RR-TB, HCV, HIV & mental health

Participants relied on providers for medical advice, but in several instances, there was little to no information given about managing their comorbidities and their impact on TB outcomes. For example, participants who smoked tobacco did not receive meaningful support to reduce or stop smoking during TB treatment, and most participants with diabetes received limited guidance on managing their blood sugar levels. One participant, whose treatment for DS-TB failed, believed he developed MDR-TB due to poor management of his diabetes.

I didn’t get any explanation at the health facility that the diabetes may become a problem…[the doctor] knew I had diabetes, but the insulin injection was not given to me…[MDR-TB] could have been avoided if I knew that I had to keep the diabetes under control. I’m very disappointed that there was no information about that. – MDR/RR-TB, diabetes

Poor linkage to care for identified comorbidities could compromise TB treatment, as experienced by several of our participants.

I took my drugs properly for four months. After four months…I went back to my former habits drinking alcohol…the [TB] drugs I have, it will only last me about three to four days. What I did, I broke the drugs into pieces in order to push me some few days ahead because I had no time to go to the clinic to get the drugs. I thought I was recovered, I thought I was healed. Then I became very sick, very, very sick. – DS-TB, HIV, substance use

Although some participants eventually did receive the comorbidity care they needed—often after a major incident such as suicidal ideation or substance use relapse—many did not. Several thus recommended greatly improving linkage to appropriate and affordable care, or integrated support, for their comorbidities.

For those with comorbidities like me, and especially those who cannot afford the metformin, the insulin, or the maintenance [therapy], maybe help from somebody aside from free TB meds. Their maintenance [therapy should] also be free, so that there is no reason for them to not finish their [TB] treatment because they cannot afford to buy…—MDR/RR-TB, diabetes

### Tailored counselling and peer support

Counselling for TB and comorbidities was a third priority. Participants found counselling specific to their comorbidities to be highly beneficial. For example, a participant facing depression due to social isolation received counselling to help talk to her family and friends about TB; another participant with diabetes learnt the importance of controlling her blood sugar levels; and some participants were likewise enabled to reduce their alcohol and drug use even in the absence of medical intervention. Other reported benefits of counselling included increased treatment motivation, confidence and knowledge.

I was really in a really bad place, and when these people [at an NGO] appeared that started helping me, I asked myself, what have I done, what good have I done that I deserve these people to come help me. And at that time, I started to feel that I needed to do this, and I can do this. –DS-TB, diabetes & mental health

Participants also highly valued guidance from peers. At times, peer supporters were the only persons in whom they confided.

Only the people with their own experience can understand …how it will affect your family, what are the fears, and how to work with the fears, what are the personal experiences. Because if the person is a professional social worker, the person cannot have the same understanding of what it’s like to have depression, to have loss of hair, or be afraid to tell the relatives. There is more trust with somebody who has survived it. –MDR/RR-TB, HIV, mental health & substance use

Peers provided helpful advice for managing comorbidities, such as accessing and handling medications, recovering from substance use disorders and dealing with suicidal ideation. This was crucial for participants who felt neglected by their TB care providers. For example, one participant only started to attend to his diabetes properly after connecting to a peer; and another was only able to cope with side effects from lupus after receiving peer advice.

### Coordinate and consolidate care for TB and comorbidities

A final priority was the involvement of people with TB comorbidities in decisions related to service or care coordination. Participants described various care-seeking behaviours for their comorbidities. Save for one participant whose TB and diabetes were managed by a primary care provider, all others sought care from multiple providers, often at different facilities. This posed a burden on participants’ time and financial resources.

People like me, we’re not in the condition to have the time or money to go. So, one day I would have an appointment with my infectious disease doctor, another day with my TB doctor, and then another time for psychiatry…so it was like three different trips and it’s very hard when you’re vulnerable. –MDR/RR-TB, HIV & mental health

Most participants preferred to receive care from as few providers and facilities as possible, but with certain conditions on who provided the care. If a participant had a trusting relationship with a provider, then they seemed more likely to appreciate having that one provider manage their TB and comorbidities, particularly for mental health problems that were believed to be precipitated by TB.

If your doctor is good then you don’t need any other counsel. If she knows about the disease, about the depression, about the whole of the person…then there is no need for anybody else. –DS-TB, mental health

However, if a participant did not have a strong relationship with any one provider, or if they had a comorbidity that they perceived required specialised skills, then they were likely to opt for multiple, well-coordinated providers.

The doctors were well coordinated because usually after I go see the MDR-TB doctor, I will get the script and the script is also sent to the lupus doctor. So, after the lupus doctor reads the script, the doctor will write medicines for my lupus. I found there was nothing wrong with the coordination [of] both treatments…The doctors have different specializations, so you cannot use one doctor for both illnesses. –MDR/RR-TB, lupus

Participants highlighted the importance of well-coordinated care in the presence of multiple providers, including having individualised treatment regimens to avoid drug interactions and adverse events. Otherwise, they were at risk of receiving incomplete or conflicting recommendations.

I saw the nutritionist on Tuesday for diabetes, and she was saying my glucose is high, and she will give me this diet…And then I saw the TB doctor, [who says] my lung is damaged and I need to eat well, get good nutrition. I didn’t know how to go about it. –DS-TB, diabetes & mental health

## Discussion

People-centred care requires addressing the multiple needs, values and priorities of affected people, alongside identifying the interventions and strategies most likely to facilitate positive clinical outcomes.^22^ At the time of development of WHO’s *Framework for collaborative action on TB and comorbidities*,^10^ few studies described firsthand experiences or perspectives of people affected by TB and a comorbid condition other than HIV,^23^,^25^ although more have been published since.^26^,^38^ By understanding the perspectives of people with TB around the world who were concurrently affected by a range of other health issues, this study identifies a set of priorities—relevant across all six WHO regions—that were included in the *Framework*.

First is early identification, via screening interventions, to facilitate self-disclosure of all comorbidities. All participants believed their TB care providers should have complete knowledge of their comorbidities, although in hindsight for some. As one participant succinctly put it: “Life depends on whether the provider has the full picture.” In the context of HIV-associated TB, most national HIV and TB programmes have implemented bidirectional screening interventions to enable early diagnosis, TB prevention and TB/HIV co-management as applicable.^6^ However, equivalent strategies to systematically screen and identify other comorbidities are inadequately (eg, mental health co-interventions^9^) or rarely adopted.^10^ Our study found that people with TB faced challenges in disclosing their comorbidities owing to a lack of patient/provider awareness of their importance, as well as concerns of stigma, discrimination and abandonment. Other studies found similar challenges among people with TB when disclosing their mental health problems; that is, that acceptability towards mental health screening and counselling depends on providers’ personality traits, and respectful and trusting provider relationships, over specialised skillsets.^30^,^32^ Thus, screening strategies should emphasise the need to increase awareness about the relevance of comorbidities in TB, as well as to develop reciprocal trust between people in TB care and their providers,^39^ without which disclosure will remain contentious particularly for conditions associated with stigma, such as mental health and substance use disorder.^2430^,^32^ Delayed or non-identification of comorbidities could have deadly consequences, most notably among people experiencing suicidal ideation, which several participants alluded to during interviews. Eliciting information on comorbidities requires trust-building, initiative and persistence, as well as empathetic communication, on the part of TB care providers.^40 41^

Second, once a comorbidity is identified, linkage to care must be assured. Participants described several barriers to accessing care for their comorbidities; even diagnosed comorbidities remained untreated. These access barriers likely contributed to negative consequences for TB, such as acquired drug resistance, and for their comorbidity, such as substance use relapse. Although all study participants received TB treatment, the majority could not access treatments for identified comorbidities. This differential accessibility has been documented among people affected by diabetes mellitus in Tanzania^26^ and tobacco or substance use in India.^23 27^ Systems-level improvements are needed to establish robust referral and linkage processes, train TB care providers in comorbidity screening and care, and strengthen primary healthcare for integrated service delivery, which has demonstrably improved outcomes in TB-HIV comorbidity.^6 33 42^

Third is counselling and peer support to allay concerns about stigma and promote health care-seeking. In the absence of counselling and psychosocial support, several participants experienced interruptions to their treatment, worsened comorbidities and relapses in substance use. This was similarly found by Li *et al*^24^ in a study in China, where people who used drugs interrupted TB treatment due to concerns about criminal prosecution, stigma and discrimination. On the other hand, in Belarus^28^ and South Africa,^29^ people with MDR-TB and substance use who received counselling found that it helped foster their motivation, confidence and capacity to improve health outcomes. These studies show that trained counsellors can build trust and cover crucial gaps in social support and health literacy about TB and comorbidities. Although evidence on the effectiveness of peer support on TB outcomes is still limited,^43^,^45^ peer-based interventions have improved outcomes among people with diabetes,^46^ mental health,^47 48^ and substance use disorders^49^ and HIV.^50^ In our study, nearly all participants sought advice from peers, with some opening up and disclosing exclusively to peers, despite efforts made by their medical team to provide this advice, highlighting the value of peer supporters in comorbidity-specific social support.

Finally, services for TB and comorbidities must be holistically coordinated with direct input from patients. Study participants often sought care from multiple providers at multiple facilities, placing avoidable demands on their time and resources and leading to conflicting treatment advice. In a study from South Africa, people affected by TB and a range of chronic diseases, including HIV, reported experiencing poorer care and communication, stigma and loss of trust and respect, when they switched from receiving integrated (single provider) care for their comorbidities, within decentralised facilities, to receiving care from multiple providers at a centralised facility.^33^ Our study participants revealed a general preference to receive services for TB and comorbidities at the same time and location, but not necessarily a single provider; some valued non-TB providers for comorbidities that were expected to be chronic or requiring specialised training. Individuals with multiple stigmatised comorbidities—such as HIV, mental health conditions and/or substance use—often experience compounded stigma, isolation and psychological distress, which can intensify when care is fragmented or poorly coordinated.^51 52^ A recent meta-analysis found that people with TB with comorbid mental health and chronic illnesses had significantly elevated rates of suicidal ideation and attempts, underscoring the urgent need for integrated, multi-pronged approaches to care.^53^ Inattention to these preferences may discourage some people with TB from revealing and accessing care for their comorbidities, with potential impacts on their TB outcome.

This research has several limitations. First, we relied on self-reported comorbidities; thus, participants might either hide certain comorbidities or report comorbidities that were not clinically diagnosed. However, our goal is to reveal the preferences and values of people affected by TB and comorbidities, that is, *their* experiences from *their* perspective, which is not compromised by using a self-report approach. Second, we used a referral-based snowball sampling approach to recruit our participants through community-based NGOs and research partners. Although this might over-represent individuals connected to specific TB service and survivor networks, our strategy allowed us to effectively reach marginalised populations with stigmatising conditions like substance use and mental health issues by leveraging established trust relationships. Indeed, our purposive sampling approach was designed with the aim of identifying priorities among people affected by TB that are relevant across all WHO regions, all genders and diverse comorbidities. However, due to the time-limited nature of this study, we restricted our sample size to 24 and included participants whose TB treatment started as early as 2015. While there have been several new recommendations issued by WHO since this time, the provision of services to improve collaborative, person-centred services for people with TB and non-HIV comorbidities may not have changed so quickly, highlighting the need for WHO’s new *Framework* and the rationale for our sampling framework.^10 14^

Notwithstanding these limitations, we were able to identify points of convergence across the lived experiences of people with multiple comorbidities from different WHO regions, while also revealing divergent experiences associated with different conditions that caution against a one-size-fits-all approach. Finally, a primary focus on disseminating findings to stakeholders engaged in developing the *Framework* and providing more immediate guidance to programmes delayed study publication. The core research principles of ethics, rigour and reciprocity led us to bring these data to the scientific forum. Our study provides a framework for future research investigating person-centred approaches that are specific to comorbidities, gender and context, to improve care and management of TB-related comorbidities.

## Conclusions

By spotlighting the perspectives of people with TB who were concurrently affected by a range of co-occurring conditions, from many regions, this study identifies a set of first principles crucial for people-centred care for TB and comorbidities. The study explicitly captures people’s opinions on preferred approaches to screen, treat and manage comorbidities without limiting the exploration to specific, individual comorbidities, nor to direct experiences of prescribed interventions or strategies. Our study reveals critical themes from an otherwise limited body of evidence on the ‘patient voice’ or ‘patient perspective’. The findings are grounded in participants’ lived experiences of TB, revealing the challenges they face navigating multiple under-reported comorbidities and the complex intersections between conditions such as substance use, HCV and mental health. The study highlights how compounding comorbidities create differential barriers—cognitive, perceptual, programmatic and structural—that disrupt care-seeking and treatment pathways for people living with TB and multimorbidity.

TB-associated comorbidities come with their own misperceptions, stigmas, fears and programme barriers that are distinct from TB disease itself and are often exacerbated by the inattention to non-TB health needs within TB programmes. Comorbidities can precipitate new challenges in the context of TB, especially mental health issues, mandating people affected by TB to be followed up for not just one, but multiple, health conditions. While disparate comorbidities have attributes that demand unique and nuanced approaches to diagnosis, treatment and care, their acceptable management *all* rely on entrusting provider relations to secure timely identification and disclosures, linkage to care and long-term retention. Affordability, accessibility, and service consolidation and coordination are key components of an acceptable people-centred model of care, though provider respect and trust, as well as connection to peer groups, remain priorities for people affected by TB. TB programmes and providers must appreciate both the common and unique needs and priorities of people with pre-existing comorbidities that could be exacerbated by TB, as well as those who develop comorbidities as a result of TB. Person-centred approaches to TB service delivery, programme linkages and resource allocation must consider the values, needs and priorities of those who are directly affected.

## Supplementary material

10.1136/bmjopen-2025-106529 online supplemental file 1

10.1136/bmjopen-2025-106529 online supplemental file 2

## Data Availability

Data are available upon reasonable request.
