# Supplementary material for: Person-centred care for people with tuberculosis-associated comorbidities: a multi-country qualitative study
Source: BMJ Open. 2025 Nov 28;15(11):e106529. doi: 10.1136/bmjopen-2025-106529 (PMC12666104; doi:10.1136/bmjopen-2025-106529)
Supplement: online supplemental file 1 [file bmjopen-15-11-s001.pdf]

Patient perspectives of people centered care for tuberculosis and co-morbidities
Study team: Amrita Daftary and Stephanie Law, York University, Canada
Funder: WHO

Interview Guide – field version

TB

1. When were you treated for tuberculosis (TB)?
2. How was treatment provided (DOT, in hospital/home)?
3. How was your experience with TB treatment? (were you cured or treated successfully).
4. During TB treatment, were you tested or asked about other health related issues? (which ones)

Comorbidity

5. I understand you may have experienced a second health related issue while you were on TB treatment. Can you tell me a little more about that? (diabetes, smoking, depression, taking drugs, heart condition, HIV, ask if >1 issue)
6. When did it start? (before or during TB treatment, what were the signs or symptoms)
7. Did you receive a formal diagnosis for it? If so, what was the process? (walk me through your journey)
8. Did you receive any type of support or treatment for it? (from where, was it formal – via health facilities, or informal – peers and family only, or a mix)

TB + Comorbidity

9. Did your TB provider know about your other health problem? (did they ask or did you tell them)
    a. What was their suggestion or instruction for managing both? (did they offer any information or follow up)
    b. Did they ask your opinion or involve you in the decision? (would you have liked to be more/less involved)
10. Did the person/provider helping you with the other issue know you had TB? (did they ask or did you tell them)
    a. What was their suggestion or instruction for managing both? (did they offer any information or follow up)
    b. Did they ask your opinion or involve you in the decision? (would you have liked to be more/less involved)
11. Eventually, how did you manage getting both types of care at the same time – for TB and the second problem?
    a. Did you face any challenges managing both? (changes to either regimen, issues taking both sets of medicines, adherence, side effects, number of pills, going to different providers/appointments, talking about them with family or friends).
    b. How did you deal with these challenges? (any changes over time)
    c. Did you have positive/helpful experiences (help from providers or other people).
    d. Do you think some of the negative experiences could have been avoided? Please share some examples. (treatment coordination, provider attitudes, relatives' attitudes, personal socio-economic issues, etc.)

12. Do you still live with the other health problem? (does it persist, how are you managing, did it stop with TB treatment, are you cured or successfully treated, any long-term challenges).
13. Thinking back, which issue was easier to deal with – for you? Can you tell me why.

Preferences

14. One of the reasons we are interviewing you is to learn how we can better support other patients who might face similar issues in the future. What do you think would have helped to make your experience with TB and your other health problem easier?
    a. Would you have preferred to receive treatment and care in a different way? Can you give me some examples:
        i. Do you wish that the care for both conditions could have been more coordinated?
        ii. Would you have liked care to be provided at the same time and place or kept separate? Can you tell me why (and if so, which clinic/provider - TB or other) or then, why not?
        iii. Would you have preferred to have more information or counselling about treating both issues together – what would you have liked to know?
        iv. Would you have preferred to have more choice or involvement?
    b. In your opinion, what supports do you think patients need when they have TB and other health problems? (information, financial, emotional/counselling… from family, health providers, peers… in person, groups, phone, other)
        i. Any advice you would suggest for a future patient in your shoes? Or a health worker taking care of people who have these concurrent issues.

Demographics

Thank you so much for your time. The interview is nearly complete. I just have a few final questions about your personal and medical history. Please answer as best as you can. We may have discussed some of these questions earlier but please bear with me if I ask them again.

15. What race or ethnicity do you identify with?

◯ Asian
◯ Black or African
◯ Hispanic or Latino
◯ White
◯ Other (specify):

16. What gender do you identify with?

◯ Female
◯ Male
◯ Other (specify):

Patient perspectives of people centered care for tuberculosis and co-morbidities
Study team: Amrita Daftary and Stephanie Law, York University, Canada
Funder: WHO

17. What was your highest level of education?
◯ No schooling
◯ Primary school not complete
◯ Primary school
◯ Secondary school, not complete
◯ Secondary school
◯ College/University

18. Are you currently employed?
◯ No (unemployed)
◯ Yes (employed). Specify:

19. What were the dates and lengths of your past treatment/s for TB?

20. What were the outcomes of your previous treatments (Probe: treatment success, completed treatment, deemed cured by your doctor, for TB and co-morbidity)

Farewell

21. Thank you for participating in our study, and for taking the time to share your experiences and perspective. Are you interested to read the summary report once our research is completed?

◯ Yes. Means of contact: email address/whatsapp ______________________
◯ No
