# Supplementary material for: Person-centred care for people with tuberculosis-associated comorbidities: a multi-country qualitative study
Source: BMJ Open. 2025 Nov 28;15(11):e106529. doi: 10.1136/bmjopen-2025-106529 (PMC12666104; doi:10.1136/bmjopen-2025-106529)
Supplement: online supplemental file 2 [file bmjopen-15-11-s002.docx]

**Initial coding tree – TB-related comorbidities**

| **Successes** | Multidisciplinary/integrated teams; comorbidity-specific interventions; staff support; peer support; enabling/supportive provider behaviours and actions; counselling and education; access to medications; socioeconomic support; integrated screening; shared decision-making |
| --- | --- |
| **Failures** | Poor provider behaviours and attitudes; lack of socioeconomic support; lack of counselling/education; barriers to accessing treatment |
| **Missed opportunities** | Withholding/hiding comorbidity information; delayed access to comorbidity care; lack of provider initiative or openness; barriers to follow-up care |
| **Preferences** | Peer support; multidisciplinary/integrated teams; provider attitudes and training; formal interventions and treatment; access to affordable care; follow-up care and monitoring; place/location of treatment; type/number of providers; coordination of care; education/counselling |
| **Context** | Comorbidity preceded/contributed to TB; socioeconomic conditions; COVID-19 restrictions; treatment/care context (multiple providers & facilities) |
| **Substance use** | Relapses; concurrent substance use; coping mechanisms; related mental health issues; attempts at stopping/cutting down |
| **HCV** | Liver/toxicity issues; concurrent HCV treatment; untreated HCV |
| **HIV** | Integrated, centralized care; concurrent HIV treatment |
| **Diabetes** | Untreated/unmanaged diabetes; concurrent diabetes treatment; follow-up and monitoring |
| **Autoimmune** | Interaction of medications; controlling/managing comorbidity |
