# Supplementary material for: Person-centred care for people with tuberculosis-associated comorbidities: a multi-country qualitative study
Source: BMJ Open. 2025 Nov 28;15(11):e106529. doi: 10.1136/bmjopen-2025-106529 (PMC12666104; doi:10.1136/bmjopen-2025-106529)
Supplement: Supplementary file 3 [file bmjopen-2025-106529.reviewer_comments.pdf]

PEER REVIEW HISTORY

BMJ Open publishes all reviews undertaken for accepted manuscripts. Reviewers are asked to complete a checklist review form and are provided with free text boxes to elaborate on their assessment. These free text comments are reproduced below.

ARTICLE DETAILS

Title (Provisional)

Person-centred care for people with tuberculosis-associated comorbidities: a multi-country qualitative study

Authors

Law, Stephanie; Baddeley, Annabel; Carlqvist, Anna; Mavhunga , Farai; Viney, Kerri; Daftary, Amrita

---

VERSION 1 - REVIEW

**Reviewer**	**1**
**Name**	**Lestari, Bony Wiem**
**Affiliation**	**Universitas Padjadjaran**
**Date**	**23-Jul-2025**
**COI**	**None**

---

1. The research objectives have not been clearly defined, neither in the abstract nor in the main body of the article. Based on the review of the articles, three potential research objectives could be identified:

(1) To understand the perspective of health care and preferences of people with TB who concurrently have comorbidities

(2) To identify a set of healthcare priorities adopted in the WHO's framework for collaborative actions on TB and comorbidities

(3) To contribute to the development of a people-centered global framework for collaborative action on TB

Should the research objectives be interconnected, it is recommended to explicitly articulate their relationship, either in the abstract or in the main body of the article.

2. While the methodology is appropriate for the study design and generally well-described, the rationale for selecting Sekhon et al.'s (2017) healthcare acceptability framework for thematic analysis remains underdeveloped. Specifically, the articles lack:

(1) A clear rationale for choosing this framework over alternatives (if there's any)

(2) Explicit discussion of how its dimensions (e.g., burden, affective attitude, ethicality, etc.,) guided the analysis

3. Taking into account the second comment, while the authors describe their thematic analysis process (between lines 15-29 with description of "the researcher (SL) then iteratively compared, collapsed, and related the codes to develop themes – paying close attention to similarities and differences in experiences based on the participant's sex, comorbidity, and geopolitical context – and created a visual model to conceptualize the themes"), the rationale for final theme selection remains unclear. For instance:

- Why were four themes ultimately chosen? Were they the most dominant, recurrent, or analytically significant in addressing the research objectives?

- How did the healthcare acceptability framework inform theme development? Was the process purely inductive, or were themes shaped by prior theoretical expectations?

A more transparent discussion of these considerations. Furthermore, a visual representation of code-to-theme mapping, would strengthen the analytical rigor.

Reviewer **2**

Name **Sweetland, Annika**

Affiliation **Columbia University Vagelos College of Physicians and Surgeons, Psychiatry**

Date **15-Aug-2025**

COI **None**

This manuscript describes a pragmatic qualitative study designed to address a significant gap in the literature capturing patient perspectives on managing TB- comorbidities among people with TB to inform WHO's Framework for collaborative action on TB and comorbidities. The authors used convenience methods to identify a sample of 24 recent TB treatment completers with self-reported comorbidities from all six WHO regions. The authors conducted semi-structured interviews informed by an established healthcare acceptability framework to elicit perspectives about their TB treatment experience with respect to managing TB-related comorbidities and explore priorities and preferences. Interviews were conducted virtually in the preferred language of each participant. The methods are clearly described and the results are well articulated. The manuscript could benefit from several minor improvements:

1) Several participants talk about or allude to suicidality, yet this is not adequately addressed in the summary of results or discussion.

• p 6 line 53 "why should I live in this world if my family doesn't want me"

• p 7 lines 22-23, “they would ask me these questions ‘do you feel suicidal?’ I would say no, I would often lie.”),

2) page 7, line 42: suggest replacing “depressed” (in the colloquial sense) with another word like “distressed”

3) page 11, line 8: add “mental health” to HIV – plenty had been published about it prior to the development of the framework, e.g.

• Agbeko CK, Mallah MA, He B, Liu Q, Song H, Wang J. Mental Health Status and Its Impact on TB Treatment and Its Outcomes: A Scoping Literature Review. Front Public Health. 2022 May 31;10:855515.

• Acha J, Sweetland A, Guerra D, Chalco K, Castillo H, Palacios E. Psychosocial support groups for patients with multidrug-resistant tuberculosis: five years of experience. Glob Public Health. 2007;2(4):404-17.

4) The fact that several common comorbidities are also stigmatized is mentioned several times (especially HIV, mental health, substance use), but not the impact of the compounding effects/intersectionality of multiple stigmas

• P. 11, line 33

• P. 13 line 33

5) Page 12: add limitations associated with the convenience sampling strategy

---

VERSION 1 - AUTHOR RESPONSE

Reviewer: 1
Dr. Bony Wiem Lestari, Universitas Padjadjaran
Comments to the Author:
1. The research objectives have not been clearly defined, neither in the abstract nor in the main body of the article. Based on the review of the articles, three potential research objectives could be identified:
(1) To understand the perspective of health care and preferences of people with TB who concurrently have comorbidities
(2) To identify a set of healthcare priorities adopted in the WHO's framework for collaborative actions on TB and comorbidities
(3) To contribute to the development of a people-centered global framework for collaborative action on TB
Should the research objectives be interconnected, it is recommended to explicitly articulate their relationship, either in the abstract or in the main body of the article.

Response: Thank you, indeed, the study objective was to understand the "perspectives of people with lived experience of TB and its associated comorbidities " (as stated in the Abstract's *Introduction*) in order to inform the development of WHO's *Framework for collaborative action on TB and comorbidities.* We have clarified this in the *Introduction:* "At the time of its development in 2021-22, there was a paucity of primary research examining the health care perspectives and preferences of people who experienced comorbidities other than HIV during their TB treatment. Thus, we conducted the following qualitative study with TB survivors from high TB burden countries to fill this knowledge gap and inform the development of the Framework."

2. While the methodology is appropriate for the study design and generally well-described, the rationale for selecting Sekhon et al.'s (2017) healthcare acceptability framework for thematic analysis remains underdeveloped. Specifically, the articles lack:
(1) A clear rationale for choosing this framework over alternatives (if there's any)
(2) Explicit discussion of how its dimensions (e.g., burden, affective attitude, ethicality, etc.,) guided the analysis

Response: The framework was drawn on only to develop the interview guide and was not applied during analysis, we have added clarification under '2.2 Data collection': "The interview guide (**supplemental material**) drew from a healthcare acceptability framework developed by Sekhon et al. [16], specifically inquiring on several dimensions of acceptability (affective attitude, burden, ethicality), and was informed by the study team's robust experience in qualitative TB research (SL, AD) and policy development (AB, AC, FM, KV)." In our analysis section, we stated that "we performed thematic analysis to inductively develop themes."

3. Taking into account the second comment, while the authors describe their thematic analysis process (between lines 15-29 with description of "the researcher (SL) then iteratively compared, collapsed, and related the codes to develop themes – paying close attention to similarities and differences in experiences based on the participant's sex, comorbidity, and geopolitical context – and created a visual model to conceptualize the themes"), the rationale for final theme selection remains unclear. For instance:
- Why were four themes ultimately chosen? Were they the most dominant, recurrent, or analytically significant in addressing the research objectives?
- How did the healthcare acceptability framework inform theme development? Was the process purely inductive, or were themes shaped by prior theoretical expectations? A more transparent discussion of these considerations. Furthermore, a visual representation of code-to-theme mapping, would strengthen the analytical rigor.

Response: As stated in our previous response, the Sekhon's framework did not inform the analysis, analysis was purely inductive. To stay within the word count, we opted to only

generally describe our methods but direct readers to our citation (#18) for more details on how reflexive thematic analysis is conducted: "We performed reflexive thematic analysis [18] to inductively develop themes based on interview transcripts using Microsoft OneNote." Analysis was done with the research objective in mind (i.e. to understand TB survivors' perspectives and lived experiences in order to inform development of WHO's Framework). Supplemental materials include the initial coding tree for analysis, as recommended in COREQ-32, which we referenced in our paper. It would be difficult to visually map all our codes to themes, as this would require oversimplification of the process and risk misrepresentation of the analysis performed. We have thus opted to not include such a visual representation; we believe our written text in the findings section thickly describes our data and relates the data to our themes.

Reviewer: 2
Prof. Annika Sweetland, Columbia University Vagelos College of Physicians and Surgeons
Comments to the Author:
This manuscript describes a pragmatic qualitative study designed to address a significant gap in the literature capturing patient perspectives on managing TB-comorbidities among people with TB to inform WHO's Framework for collaborative action on TB and comorbidities. The authors used convenience methods to identify a sample of 24 recent TB treatment completers with self-reported comorbidities from all six WHO regions. The authors conducted semi-structured interviews informed by an established healthcare acceptability framework to elicit perspectives about their TB treatment experience with respect to managing TB-related comorbidities and explore priorities and preferences. Interviews were conducted virtually in the preferred language of each participant. The methods are clearly described and the results are well articulated. The manuscript could benefit from several minor improvements:
1) Several participants talk about or allude to suicidality, yet this is not adequately addressed in the summary of results or discussion.
• p 6 line 53 "why should I live in this world if my family doesn't want me"
• p 7 lines 22-23, "they would ask me these questions 'do you feel suicidal?' I would say no, I would often lie."),

Response: Thank you for pointing this out. We have specifically highlighted it in the *Overview* of our results, "Most reported comorbidities preceded TB diagnosis; except for mental health conditions, including suicidal ideation, which many participants attributed to TB and its associated burden." We additionally added the following text in our discussion: "Delayed or non-identification of comorbidities could have deadly consequences, most notably among people experiencing suicidal ideation, which several participants alluded to during interviews."

2) page 7, line 42: suggest replacing "depressed" (in the colloquial sense) with another word like "distressed"

Response: Thank you for this suggestion, we have made the replacement.

3) page 11, line 8: add "mental health" to HIV – plenty had been published about it prior to the development of the framework, e.g.
• Agbeko CK, Mallah MA, He B, Liu Q, Song H, Wang J. Mental Health Status and Its Impact on TB Treatment and Its Outcomes: A Scoping Literature Review. Front Public Health. 2022 May 31;10:855515.
• Acha J, Sweetland A, Guerra D, Chalco K, Castillo H, Palacios E. Psychosocial support groups for patients with multidrug-resistant tuberculosis: five years of experience. Glob Public Health. 2007;2(4):404-17.

Response: Thank you for pointing this out. We agree that plenty has been published regarding mental health & TB, however, implementation of mental health interventions remains inadequate around the world - this is highlighted by our participants where the 'screening' for mental health conditions equated to a checklist prior to any established trust or rapport that might facilitate disclosure. We have as such added the following text in our discussion: "However, equivalent strategies to systematically screen and identify other comorbidities are inadequately (e.g. mental health co-interventions [39]) or rarely adopted [10]."

4) The fact that several common comorbidities are also stigmatized is mentioned several times (especially HIV, mental health, substance use), but not the impact of the compounding effects/intersectionality of multiple stigmas
• P. 11, line 33
• P. 13 line 33

Response: Thank you for highlighting this shortcoming - we have added the following text to our discussion: "Individuals with multiple stigmatized comorbidities – such as HIV, mental health conditions and/or substance use – often experience compounded stigma, isolation, and psychological distress, which can intensify when care is fragmented or poorly coordinated [53, 54]. A recent meta-analysis found that people with TB with comorbid mental health and chronic illnesses had significantly elevated rates of suicidal ideation and attempts, underscoring the urgent need for integrated, multi-pronged approaches to care [55]."

5) Page 12: add limitations associated with the convenience sampling strategy

Response: We added the following limitation to our discussion: 'Second, we used a referral-based snowball sampling approach to recruit our participants through community-based NGOs and research partners. Although this might over-represent individuals connected to specific TB service and survivor networks, our strategy allowed us to effectively reach

marginalized populations with stigmatizing conditions like substance use and mental health issues by leveraging established trust relationships."

VERSION 2 - REVIEW

**Reviewer**	**1**
**Name**	**Lestari, Bony Wiem**
**Affiliation**	**Universitas Padjadjaran**
**Date**	**30-Oct-2025**
**COI**	

The authors are to be commended for their revisions. The manuscript has been significantly improved and clarified in response to the previous round of feedback. The study provides a valuable and pragmatic contribution to the field by centering the patient perspective to inform and enhance the WHO TB-comorbidity framework. The methodology is sound and well-described, the results are nuanced and presented with clarity, and the discussion effectively synthesizes the findings to achieve the study's objective.

The manuscript is in good shape and is suitable for publication in its current form. Below are minor, optional suggestions to further strengthen an already compelling paper.

Minor Suggestions for Improvement:

1. Strengthening the Conclusion: The first paragraph of the conclusion effectively highlights the conceptual imperative of incorporating patient voices in designing patient centered care. To make it even more impactful, I suggest tightening the first paragraph lines 13-18 (p.40) to reduce some redundancy with later points (lines 48-51, p.40) and streamlining sentences in lines 27-31 (p.40). This will sharpen the focus on the core evidence and make the final message more forceful.

2. Future Research Directions: The paper would benefit from a more explicit discussion of future research pathways. Briefly outlining how other researchers could build upon this work. For instance, extending these findings to different populations or by addressing the specific limitations noted in the study would provide a clearer roadmap for the scientific community and underscore the study's broader relevance.

VERSION 2 - AUTHOR RESPONSE

Reviewer: 1
Dr. Bony Wiem Lestari, Universitas Padjadjaran
Comments to the Author:
The authors are to be commended for their revisions. The manuscript has been significantly

improved and clarified in response to the previous round of feedback. The study provides a valuable and pragmatic contribution to the field by centering the patient perspective to inform and enhance the WHO TB-comorbidity framework. The methodology is sound and well-described, the results are nuanced and presented with clarity, and the discussion effectively synthesizes the findings to achieve the study's objective.

The manuscript is in good shape and is suitable for publication in its current form. Below are minor, optional suggestions to further strengthen an already compelling paper.

Minor Suggestions for Improvement:

1. Strengthening the Conclusion: The first paragraph of the conclusion effectively highlights the conceptual imperative of incorporating patient voices in designing patient centered care. To make it even more impactful, I suggest tightening the first paragraph lines 13-18 (p.40) to reduce some redundancy with later points (lines 48-51, p.40) and streamlining sentences in lines 27-31 (p.40). This will sharpen the focus on the core evidence and make the final message more forceful.

Response: Thank you for the suggestion, we have removed the first (redundant) sentence from the conclusion and improved/streamlined lines 27-31.

2. Future Research Directions: The paper would benefit from a more explicit discussion of future research pathways. Briefly outlining how other researchers could build upon this work. For instance, extending these findings to different populations or by addressing the specific limitations noted in the study would provide a clearer roadmap for the scientific community and underscore the study's broader relevance.

Response: We have added the following to the end of our discussion: Our study provides a framework for future research investigating person-centred approaches that are specific to comorbidities, gender, and context, to improve care and management of TB-related comorbidities.

Reviewer: 1
If you have selected ‘Yes’ above, please provide details of any competing interests.: Not applicable

---
